# Insights into the Drought and Heat Avoidance Mechanism in Summer-Dormant Mediterranean Tall Fescue

**DOI:** 10.3389/fpls.2017.01971

**Published:** 2017-11-17

**Authors:** Ali M. Missaoui, Dariusz P. Malinowski, William E. Pinchak, Jaime Kigel

**Affiliations:** ^1^Institute of Plant Breeding Genetics and Genomics, The University of Georgia, Athens, GA, United States; ^2^Texas AgriLife Research and Extension Center, Vernon, TX, United States; ^3^The Robert H. Smith Institute of Plant Sciences and Genetics in Agriculture, Hebrew University of Jerusalem, Rehovot, Israel

**Keywords:** summer-dormancy, vernalization, drought tolerance, rubidium, determinacy, tall fescue

## Abstract

Summer dormancy is an evolutionary response that some perennial cool-season grasses adopted as an avoidance strategy to escape summer drought and heat. It is correlated with superior survival after severe summer droughts in many perennial grass species originating from Mediterranean environments. Understanding the genetic mechanism and environmental determinants of summer dormancy is important for interpreting the evolutionary history of seasonal dormancy and for the development of genomic tools to improve the efficiency of genetic selection for this important trait. The objectives of this research are to assess morphological and biochemical attributes that seem to be specific for the characterization of summer dormancy in tall fescue, and to validate the hypothesis that genes underlying stem determinacy might be involved in the mechanism of summer dormancy. Our results suggest that vernalization is an important requirement in the onset of summer dormancy in tall fescue. Non-vernalized tall fescue plants do not exhibit summer dormancy as vernalized plants do and behave more like summer-active types. This is manifested by continuation of shoot growth and high root activity in water uptake during summer months. Therefore, summer dormancy in tall fescue should be tested only in plants that underwent vernalization and are not subjected to water deficit during summer months. Total phenolic concentration in tiller bases (antioxidants) does not seem to be related to vernalization. It is most likely an environmental response to protect meristems from oxidative stress. Sequence analysis of the *TFL1* homolog *CEN* gene from tall fescue genotypes belonging to summer-dormant and summer-active tall fescue types showed a unique deletion of three nucleotides specific to the dormant genotypes. Higher tiller bud numbers in dormant plants that were not allowed to flower and complete the reproductive cycle, confirmed that stem determinacy is a major component in the mechanism of summer dormancy. The number of variables identified in these studies as potential players in summer dormancy in tall fescue including vernalization, *TFL1/CEN*, water status, and protection from oxidative stress are a further confirmation that summer dormancy is a quantitative trait controlled by several genes with varying effects and prone to genotype by environment interactions.

## Introduction

Numerous ecotypes of cool-season perennial grasses originating from the Mediterranean Basin of southern Europe and northern Africa, and Mediterranean environments of California exhibit summer-dormancy, primarily in response to increasing day length and probably high temperatures (Laude, 1953; Vegis, 1964; Ofir and Kigel, 2003). Summer dormancy in cool-season perennial grasses is an endogenously controlled process involving the cessation or reduction of leaf growth, the complete or partial senescence of herbage and, in some cases, the endogenous dehydration of meristems (Volaire and Norton, 2006). Summer dormancy is induced independently from soil moisture, even though it can be accelerated by drought (Norton et al., 2006b). The process is most likely initiated under short photoperiods and relatively low temperatures during winter. Orchardgrass (*Dactylis glomerata* L.) and tall fescue [*Lolium arundinaceum* (Schreb.) S.J. Darbyshire] must be exposed to these conditions for the summer dormancy trait to be fully expressed under the subsequent long photoperiods and high temperatures of summer (Norton et al., 2006a,b). Summer-dormant cool-seasonal perennial grasses produce dormant regenerating buds at tiller bases during spring, from which growth resumes in response to increased water availability (i.e., rainfall) and decreasing temperatures in autumn. The temporary growth suspension is an evolutionary response that these grasses adopted as an avoidance strategy to escape summer drought and heat (Laude, 1953; Ofir and Kerem, 1982; Oram, 1983; Volaire and Thomas, 1995). In some species (i.e., *Phalaris aquatica* L. and *Hordeum bulbosum* L.), surviving perenniating buds are associated with corms produced at the base of flowering tillers. In these species, the onset of summer dormancy may be closely associated with timing of flowering (Ofir and Kigel, 2003).

Differences between dormancy types may be gradual and depend on responsiveness to inducing and synergistic factors (i.e., day length, high temperature, soil moisture status), depth of dormancy, and kinetics of dormancy relaxation during summer (Kigel et al., 2009). After dormancy relaxation, growth of regenerating buds is arrested during summer by lack of water. It is conceivable that summer-dormant cool-season grasses differ in the duration of dormancy (fast vs. prolonged relaxation from dormancy) and, hence in their drought tolerance and response to summer rainfall events (Norton et al., 2008). Summer dormancy has a continuous expression in cool-season perennial grasses as described by an index of summer dormancy (SDI). This index is based on comparing irrigated summer herbage yield of a cultivar with that of a high, summer-yielding, non-dormant control cultivar (Norton et al., 2008, 2009). The SDI values may vary from zero (summer-active types) to 10 (summer-dormant types). As a result, some ecotypes (such as for tall fescue) may express partial summer dormancy as measured by SDI values of 2 to 6 (Malinowski et al., 2009).

During dormancy and growth cessation, basal meristems in the plant crown perceive eliciting signals and become inactive even when growth conditions are favorable (Rohde and Bhalerao, 2007). This involves changes in the expression levels of various genes and growth hormones. Auxin response factors (ARFs) were reported to activate the transcription of auxin-responsive genes involved in growth responses, which are also related to seasonal dormancy (Shim et al., 2014). Flowering time regulation has been linked with seasonal growth cessation, illustrating that regulatory hierarchies of developmental processes might be recruited into the control of dormancy processes. Overexpression of *FT1* and *CO* homologues of poplar [*Populus balsamifera* L. ssp. *trichocarpa* (Torr. and A. Gray ex Hook.) Brayshaw] lead to growth cessation in aspen (*Populus tremuloides* Michx.) exposed to short photoperiod (Böhlenius et al., 2006). Effects of the *FRIGIDA* gene at flowering time was also well documented in *Arabidopsis* including the positive influence of *FT* expression (Lee and Amasino, 1995; Michaels and Amasino, 2001; Caicedo et al., 2004; Shindo et al., 2005; Korves et al., 2007). Molecular models for photoperiod and temperature associated dormancy suggest that short day and short-term exposure to low temperatures induce dormancy, and the process is regulated by the *CO/FT* module and *DAM* gene expression (Horvath, 2009; Campoy et al., 2011). Other genes known for their involvement in the control of seasonal dormancy include circadian clock regulators, such as *GIGANTEA (GI)* and *CIRCADIAN CLOCK ASSOCIATED1* (Cao et al., 2005; Oliverio et al., 2007; Chiang et al., 2009; Penfield and Hall, 2009; Ibanez et al., 2010), *FLOWERING LOCUS T* (*FT*) and the *CO*/*FT* complex (Pin and Nilsson, 2012), *CENTRORADIALIS1* (*CEN1*/ *TERMINAL FLOWER1* (*TFL1*) (Mohamed et al., 2010), as well as *dormancy-associated MADS-box* factors (Horvath et al., 2010).

*TFL*1-like genes are highly conserved in plants and are believed to help maintain meristem indeterminacy. Determinacy is an agronomically important trait associated with domestication of crop species (Tian et al., 2010). The meristems in determinate plants cease vegetative growth and switch to reproductive inflorescence following exposure to flowering inducing photoperiod (Bernard, 1972), similar to the dormancy phenotype. Ectopic expression of *TFL1*-like genes *Zea* Centroradialis (*ZCN*1-*ZCN6*) in maize modifies flowering time and inflorescence architecture through maintenance of the indeterminacy of the vegetative and inflorescence meristems (Danilevskaya et al., 2010).

In a previous study, we explored the association between summer dormancy phenotypes and 23 candidate genes using 52 dormant and non-dormant tall fescue accessions. The 52 dormant and non-dormant accessions were selected based on the evaluation of 218 tall fescue accessions for summer dormancy in field conditions for 2 years, in addition to assigning surrogate phenotype scores based on the ratio of seed germination at 30°C and 20°C under 24 h photoperiod in growth chamber (Ding and Missaoui, 2016). The results showed significant associations between field phenotypic scores and five markers that originated from the genes CONSTANS and TERMINAL FLOWER, suggesting that summer-dormant tall fescue plants are most likely using stem determinacy as a mechanism to initiate dormancy (Ding and Missaoui, 2017).

In this publication, we present results of a series of experiments based on the following hypotheses: (1) Summer dormancy mechanism in tall fescue relies on specific morphological (i.e., production of flowering tillers, cessation of shoot growth and resulting summer dormancy index), physiological (i.e., reduction in root activity and water uptake as measured by rubidium concentration in shoots), and biochemical adaptations (i.e., protection of shoot meristems from oxidative stress as measured by total phenolic compound concentrations); (2) Genes regulating stem determinacy (transition from vegetative to reproductive stage) might be directly involved in triggering the onset of summer dormancy in tall fescue.

Understanding the genetic basis and environmental determinants of summer dormancy is essential for interpreting the evolutionary history of seasonal dormancy and for the development of genomic tools to improve the efficiency of genetic selection for this important stress avoidance mechanism.

## Materials and Methods

### Vernalization and Drought Response in Summer-Active and Summer-Dormant Tall Fescue

In a series of greenhouse experiments conducted during March – October 2006 and 2007, we measured selected physiological and biochemical traits in summer-dormant and summer-active tall fescue plants in response to vernalization and a gradually imposed soil water deficit stress. The experiments consisted of three treatments including summer dormancy (dormant and non-dormant fescue types), vernalization (vernalized and non-vernalized plants), and soil moisture deficit stress (non-stressed control and stressed plants). Each treatment was replicated four times. Summer-dormant tall fescue types were represented by the cultivars Flecha, Prosper, and an experimental line TX06V-BEF. Summer-active types were represented by the cultivars Barcarella, Drover, and Kentucky 31. Each tall fescue cultivar was represented by 16 plants. All tall fescue entries were free of the mutualistic endophyte *Epichloë cenophiala* (Leuchtmann et al., 2014). Two groups of plants were produced: vernalized and non-vernalized. Vernalized plants were obtained from seeds planted in late October in the field and exposed to typical winter temperatures at Vernon, TX (United States). Non-vernalized plants were obtained from seeds planted in the greenhouse in early January and maintained under ambient light conditions and temperature of 24/15°C day/night. Plants with similar weights (5 g DM) were individually planted individually planted on March 1, in 3.8 dm^3^ pots filled with 2.8 kg dry Miles fine sandy loam (fine-loamy, mixed, Thermic Udic Paleustalfs; 12% of maximum water holding capacity). Each pot was watered with588 ml water to achieve 90% soil water content. The soil surface was covered with 450 g fine gravel to reduce evaporation. During the establishment phase (1 March – 31 May), all pots were individually weighed daily and water was added to maintain 90% soil water content. Plants were fertilized once a week with 100 ml of Miracle Gro^®^ commercial fertilizer dissolved at the rate of 1.3 g dm^-3^. Temperature in the greenhouse was maintained at 34/24°C day/night with ambient light conditions.

On 1 June, shoots of all plants were clipped to 5 cm height, oven-dried at 65°C for 48 h, then weighed. Half of the plants were exposed to a gradually imposed soil moisture deficit stress cycle ranging from 90 to 60%, 30, and 15% soil water content. Each soil moisture deficit stage lasted for 7 days to a total of 4 weeks per stress cycle. Pots were weighed every day and water added as needed to ensure the target stress levels. At the end of the stress cycle, plants were re-watered to 90% soil water content and allowed to regrow for 4 weeks (recovery phase from stress). These plants were exposed again to a second cycle of a gradually imposed soil moisture deficit stress lasting 4 weeks, re-watered and allowed to regrow for another 4 weeks. Control plants were maintained at 90% soil water content during the entire course of the experiments.

Flowering tillers were defined as tillers with a visible or developing inflorescence and counted at each stage of the soil moisture stress cycle. For the purpose of this study, we present the percentage of total flowering tiller number to the total number of all tillers produced on the plant during June – September.

Chlorophyll content in leaf blades was measured at the 3rd fully matured leaf from the top of the tiller, on leaf segments about 5 cm away from the culm. Chlorophyll was measured using a CCM-200 Plus chlorophyll content meter and expressed as chlorophyll content index (CCI) units.

The concentration of the trace element rubidium (Rb) was used here to monitor water uptake efficiency by roots (Messerli, 1997). Five ml of rubidium chloride solution at the concentration of 1 g dm^-3^ was injected into the soil at 10 cm depth at the beginning of the 1st soil moisture deficit cycle (6 June). Prior to injection of Rb, shoots were analyzed for naturally occurring Rb concentration (background) that ranged from 0.0001 to 0.0009 mg g^-1^ DM. Shoots were harvested at 5 cm height at the end of the 1st soil moisture deficit cycle (7 July). Concentration of Rb in plant material was measured using atomic absorption flame spectrophotometry in a commercial laboratory.

Antioxidants, including phenolic compounds, may protect growing meristems from oxidative stress during summer drought (Cui et al., 2006; Malinowski et al., 2009). Total phenolic concentrations were measured in tiller bases collected at the beginning and the end of the 1st imposed soil moisture deficit, and at the end of the 2nd moisture deficit. Tiller bases (1.5 cm long) were frozen in Ziploc bags at -25°C within 30 min after collecting, and remained frozen until the analysis. Concentrations of total phenolics were determined using the modified Price and Butler method (Waterman and Mole, 1994). The method quantifies the total concentration of phenolic hydroxyl groups present in the assayed extract. Approximately 3 g of frozen tiller bases were homogenized in 50 ml ethanol:water (50:50 by volume) for 30 s and subsequently filtered using Whatman No. 1 filter paper. Exactly 1 ml of the supernatant and 49 ml of distilled water were added to a 150 ml flask and mixed thoroughly. Ferric chloride (3 ml) was added to flasks containing the diluted supernatant and flasks with blank (water) and standards. Three minutes later, 3 ml potassium ferricyanide were added to the mixture. After 15 min of incubation in darkness, the absorbance of samples and standards against the blank was determined at 720 nm using a Helios UV-Visible Spectrophotometer (Thermo Fisher Scientific Inc., Waltham, MA, United States). Standards at a range of concentrations of 0.0–0.2% were prepared with tannic acid. The total phenolic concentration was calculated from a calibration curve and expressed on dry weight basis.

Measuring shoot growth during summer under non-limiting soil moisture as proposed by Norton et al. (2008) was found to be a good measure of summer dormancy expression in cool-season grasses (Malinowski et al., 2009). Summer dormancy index (SDI) was calculated as a ratio of shoot DM of a given tall fescue accession produced during summer (June–August) to shoot DM of a maximum-yielding accession using the following equation (Norton et al., 2008):

SDI = (1 - shoot DM of accession Ashoot DM of maximum-yielding accession B) × 10

The experiments were set up as completely randomized designs. In each experiment, treatments were tall fescue summer dormancy type (dormant or non-dormant), vernalization status (vernalized or non-vernalized), and soil moisture deficit stress (non-stressed control or stressed plants)., Each treatment was replicated four times. Summer dormancy, vernalization, and soil moisture stress were considered fixed effects, and tall fescue cultivars was were considered random effects. For the purpose of analyzing SDI, tall fescue cultivars with known summer-dormancy and p vernalization status were considered fixed effects and replications as random effect. A three-way ANOVA analysis of variance was performed using the Mixed Procedure of the SAS statistical package (SAS Institute, 1999). Statistical analyses were performed separately for each growing season (year) because the tall fescue cultivars evaluated varied each season. Prior to analysis of variance, data were transformed for percentage values of flowering tillers (arcsin transformation) and chlorophyll, rubidium and total phenolic contents, and summer dormancy index (ln transformation) to ensure normal distribution of data. Means between summer dormancy type, vernalization type, and soil moisture stress status treatments were compared by the pairwise multiple comparison test (LSD). Significance of means was declared at *P* = 0.05.

### Validation of the Involvement of *TFL1/CEN* Gene Family in Summer Dormancy

#### *TFL*1 Sequence Search, Alignment, and Polymorphism Identification

To validate the hypothesis that genes underlying stem determinacy might be involved in the mechanism of summer dormancy (Ding and Missaoui, 2017), we conducted a search in the National Center for Biotechnology Information (NCBI) databases for published *TFL*1 genes from the *Lolium* genus. The query resulted in a sequence of 954 bp from *Lolium perenne* terminal flower 1-like protein (*TFL*1) mRNA (Locus AF316419). This sequence showed 91% similarity to a predicted *Brachypodium distachyon* Centroradialis (*CEN*)-like protein 2 (LOC100844906), mRNA. *CEN* is a floral developmental identity-determining gene, also known as terminal flower 1. Blast (NCBI BLAST, Bethesda, MD, USA) queries of the *Lolium perenne TFL*1 nucleotide sequence were conducted in the genus *Festuca* and *Lolium perenne* expressed sequence tags (EST), and *Brachypodium distachyon* genome sequence release. It showed 100% similarity with a *Lolium perenne CEN* gene, and 93–99% similarity to 37 *CEN* sequences from various *Festuca* species deposited in the database by Hand et al. (2010). Twenty of the sequences were from *Festuca arundinacea* (syn. *Lolium arundinaceum* (Schreb.) S. J. Darbyshire), representing seven cultivars. Three of the seven cultivars were known continental summer-active types (Ky31, Jesup, and Quantum), and two were known Mediterranean summer-dormant types (Resolute and PG4012), and two rhizomatous types (CT2093R and Torpedo II). Each genotype was represented by two to three *CEN* haplotypes. The *CEN* haplotypes from each sequence were aligned using Clustal W to generate one consensus sequence per genotype. Multiple sequence alignment was conducted in Geneious software version 7.1.2 (Kearse et al., 2012) (Clustal option), with Gap penalties of 10 and gap extension 0.1. A *CEN* sequence of annual ryegrass (*Lolium multiflorum* L.) was included as a control. Nucleotide variants including SNPs and Indels between Mediterranean, continental, and rhizomatous types were identified and compared for their specificity to each tall fescue type.

#### Phenotypic Test of the Relationship between Determinacy and Summer Dormancy

To explore the possible relationship between summer dormancy and determinacy, a greenhouse experiment was initiated in September 2014 at the University of Georgia, Athens. Seed of two summer-active genotypes (GA186 and Kentucky 31) and 2 summer-dormant genotypes [T0706-1 (a half-sib from the experimental population GA95101T) and AGRFA-126 (Ag Research New Zealand)] were planted in trays in the greenhouse and then transplanted into 4 dm^-3^ pots after 8 weeks. Twenty pots containing one plant per pot were used for each check. Greenhouse conditions were maintained at a temperature of 27/15°C (day/night) and natural light conditions. The plants were watered once a day and fertilized once a week. On 3 January, all the plants were moved outside the greenhouse and maintained under natural weather conditions for exposure to natural vernalization and photoperiod conditions. After 6 weeks, the plant were moved back to the greenhouse. One group of 10 plants for each check was allowed to grow to flowering and set seed (non-clipped treatment). The other 10 plants were kept in the vegetative stage by frequently clipping them starting at the first signs of stem elongation, and were prevented from flowering and setting seed (clipped treatment). On 3 August 2014, all the plants were removed from the pots. The roots were washed, and the number of tiller buds sprouting from the crowns was counted on each plant (**Figure 6**). The data was analyzed as a completely randomized design using the PROC GLM procedure in SAS 9.4. Separation of differences in tiller count means between the clipped and no-clipped treatments within each accession was declared significant at alpha = 0.05 using Fisher’s protected least significant difference (LSD) test.

## Results

### Vernalization and Drought Response in Summer-Active and Summer-Dormant Tall Fescue

**FIGURE 1:**
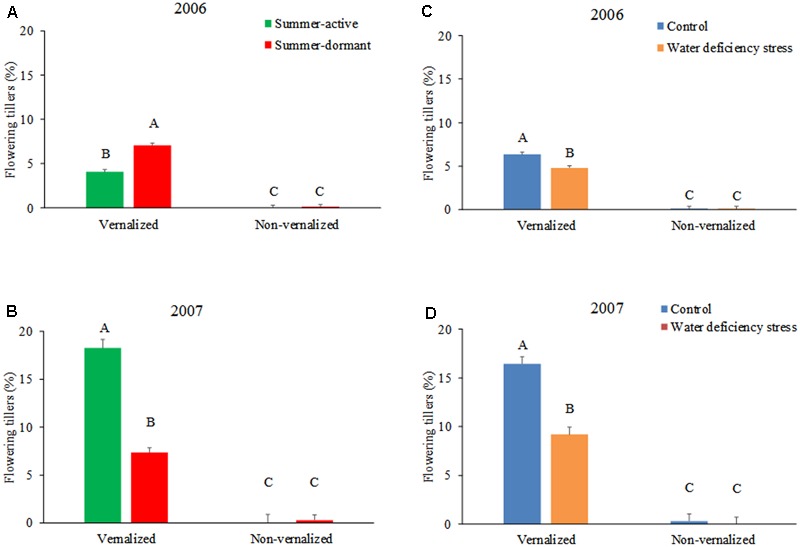
The percentage of flowering tillers of tall fescue as affected by the interactions between summer dormancy and vernalization status in 2006 **(A)** and 2007 **(B)**, and soil water deficiency stress and vernalization status in 2006 **(C)** and 2007 **(D)**. In 2006, summer-dormant entries included Flecha, Prosper, and an experimental line TX06V-BEF, while summer-active entries were Barcarella and Drover. In 2007, the summer-dormant tall fescue entries were the same as in 2006, while summer-active type was represented by Kentucky 31. Entries with the same letters are not significantly different. Bars indicate 1 SE.

The number of flowering tillers was significantly higher (*P* < 0.01) in tall fescue plants that underwent vernalization compared with that of non-vernalized plants, both in 2006 and 2007 growing season (**Figures 1A,B** and **Table 1**). Summer-dormancy type interacted with vernalization (*P* < 0.01) in determining the number of flowering tillers. In 2006, summer-dormant tall fescue cultivars produced more flowering tillers than summer-active cultivars, while summer dormancy did not affect this trait in non-vernalized plants. In contrast, summer-active tall fescue had more flowering tillers than summer-dormant tall fescue when vernalized in 2007, while non-vernalized plants did not differ as a result of summer dormancy trait. The difference in the number of flowering tillers of vernalized plants attributed to summer dormancy was likely the result of using different cultivars each year. Soil moisture deficiency stress interacted with vernalization status (*P* < 0.01) in determining the number of flowering tillers in a similar way each growing season (**Figures 1C,D** and **Table 1**). For vernalized tall fescue cultivars, the number of flowering tillers was higher for plants maintained at 90% soil water content (control) than that for plants exposed to two cycles of soil water deficiency stress. Non-vernalized tall fescue plants produced a marginal number of flowering tillers and it was not affected by soil moisture level.

**Table 1:** Analysis of variance (ANOVA) results of percentage of flowering tillers, rubidium concentration in shoots, and total phenolic concentration in tiller meristems for the randomized complete block design experiments conducted during 2006 and 2007 growing seasons.

Variable	Effect	Num DF	Den DF	*F*-value	Pr > F
Flowering tillers 2006	Vernalization (V)	1	72	29.17	0.0001
	Dormancy (D)	1	72	363.95	0.0001
	V × D	1	72	25.64	0.0001
	Soil moisture stress (S)	1	72	7.11	0.0094
	V × S	1	72	1.81	0.1829
	D × S	1	72	7.31	0.0085
	V × D × S	1	72	7.71	0.1952
Flowering tillers 2007	Vernalization (V)	1	56	52.06	0.0001
	Dormancy (D)	1	56	297.88	0.0001
	V × D	1	56	58.28	0.0001
	Soil moisture stress (S)	1	56	26.52	0.0001
	V × S	1	56	1.64	0.2054
	D × S	1	56	22.38	0.0001
	V × D × S	1	56	2.89	0.0947
Rubidium concentration	Vernalization (V)	1	72	3.97	0.0501
	Dormancy (D)	1	72	18.03	0.0001
	V × D	1	72	6.98	0.0001
	Soil moisture stress (S)	1	72	46.08	0.0001
	V × S	1	72	3.05	0.0849
	D × S	1	72	3.86	0.0499
	V × D × S	1	72	0.93	0.3387
Phenolic concentration June ‘07	Vernalization (V)	1	56	17.30	0.0001
	Dormancy (D)	1	56	0.08	0.7758
	V × D	1	56	8.31	0.0056
	Soil moisture stress (S)	1	56	0.01	0.9128
	V × S	1	56	0.09	0.7591
	D × S	1	56	0.35	0.5547
	V × D × S	1	56	0.35	0.5547
Phenolic concentration July ‘07	Vernalization (V)	1	56	29.06	0.0001
	Dormancy (D)	1	56	2.96	0.0909
	V x D	1	56	0.07	0.7987
	Soil moisture stress (S)	1	56	2.48	0.1212
	V × S	1	56	0.22	0.6435
	D × S	1	56	0.19	0.6660
	V × D × S	1	56	0.62	0.4332
Phenolic concentration Sep ‘07	Vernalization (V)	1	56	83.17	0.0001
	Dormancy (D)	1	56	5.06	0.0284
	V × D	1	56	0.09	0.7695
	Soil moisture stress (S)	1	56	0.74	0.3936
	V × S	1	56	0.05	0.8238
	D × S	1	56	1.18	0.2815
	V × D × S	1	56	1.38	0.2454

**FIGURE 2:**
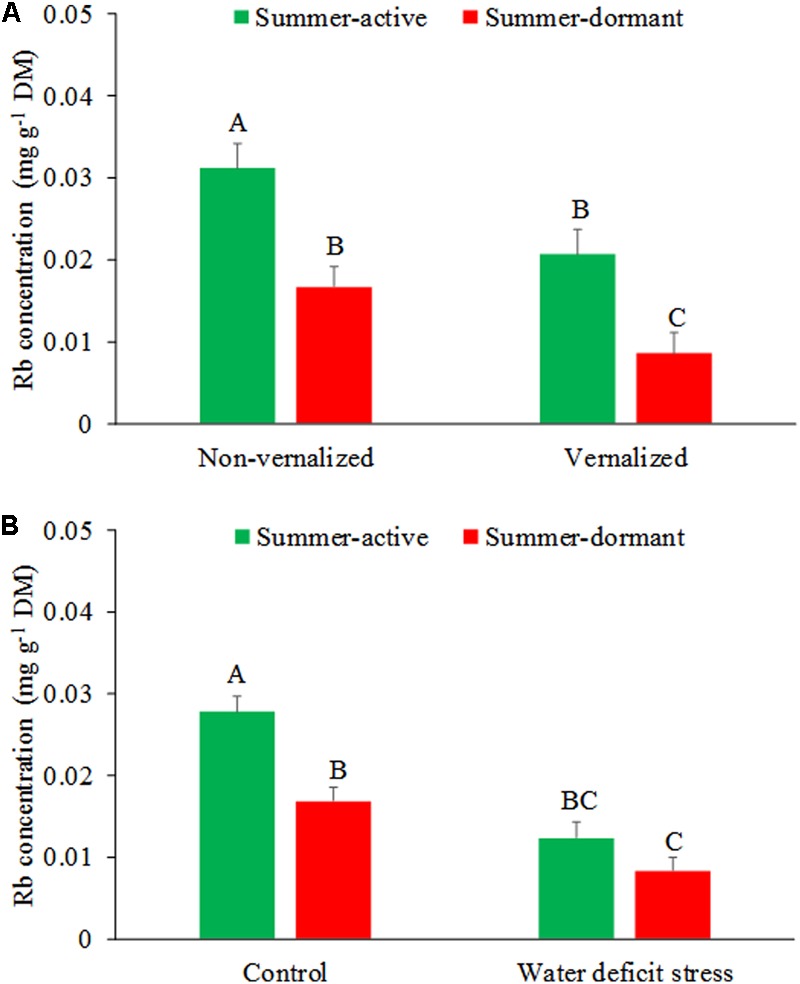
Effects of interactions between summer dormancy type and vernalization status **(A)** and soil water deficit stress and vernalization status **(B)** on Rb concentrations in tall fescue shoots during summer. Entries with the same letters are not significantly different. Bars indicate 1 SE.

Rubidium concentrations in tall fescue shoots were affected by the interaction between vernalization status and summer dormancy type (*P* < 0.01), and summer dormancy type and soil moisture stress (**Figures 2A,B** and **Table 1**). Summer-active tall fescue plants had higher Rb concentrations (*P* < 0.01) in shoots than summer-dormant plants, regardless of vernalization status. Non-vernalized summer-dormant plants had similar RB concentrations to vernalized summer-active plants (*P* > 0.05). Rubudium concentrations in plants grown at non-limiting soil moisture was higher in summer-active than summer-dormant plants, but with imposed soil water deficiency stress the difference was not significant (*P* > 0.05). Summer-dormant plants grown at non-limiting soil water did not differ in Rb concentration. This would suggest a lower water uptake by roots by summer-dormant compared to summer-active tall fescue, especially if both types were non-vernalized. This, together with the SDI data (see below), suggests that summer dormancy was not fully expressed in non-vernalized tall fescue plants when compared with vernalized plants. Similar results were also reported by Bhamidimarri et al. (2012) who concluded that summer dormancy can only be differentiated among tall fescue genotypes under conditions of long day, optimal temperature, growth non-limiting soil water availability, and in vernalized plants.

**FIGURE 3:**
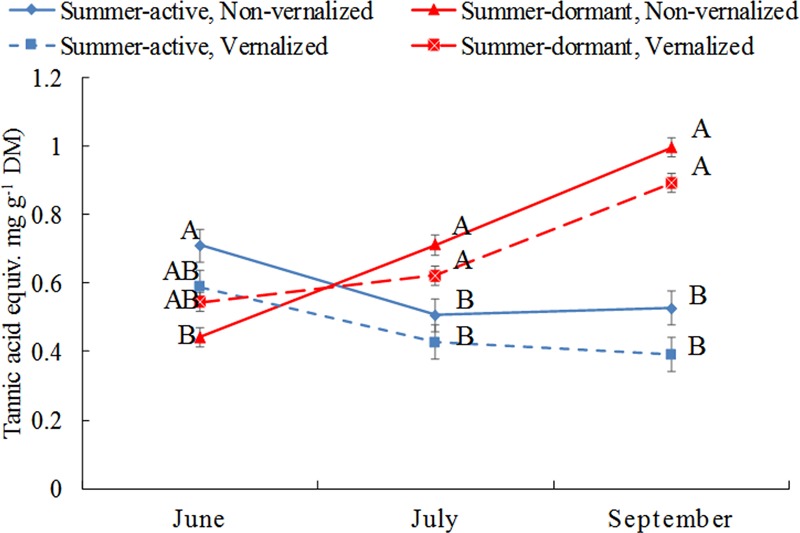
Changes in total phenolic concentration (measured as tannic acid equivalent) in shoot meristems of tall fescue in relation to summer dormancy and vernalization status in June (beginning of summer), July (mid-summer) and September (beginning of autumn). Entries with the same letters are not significantly different. Bars indicate ± 1 SE.

Total phenolic concentrations, expressed here as tannic acid equivalent, were higher (*P* < 0.01) in summer-active than these in summer-dormant tall fescue in June 2007 (beginning of Summer) if both types were not vernalized, but the difference between dormancy status was not significant in vernalized plants (**Figure 3** and **Table 1**). As the summer progressed (July and September), total phenolic concentrations in summer-dormant tall fescue plants were increasing and were higher than these in summer-active types, regardless of the vernalization status. In contrast, phenolic concentrations in summer-active tall fescue plants were initially decreasing (July) and then maintained at a constant level (September).

**FIGURE 4:**
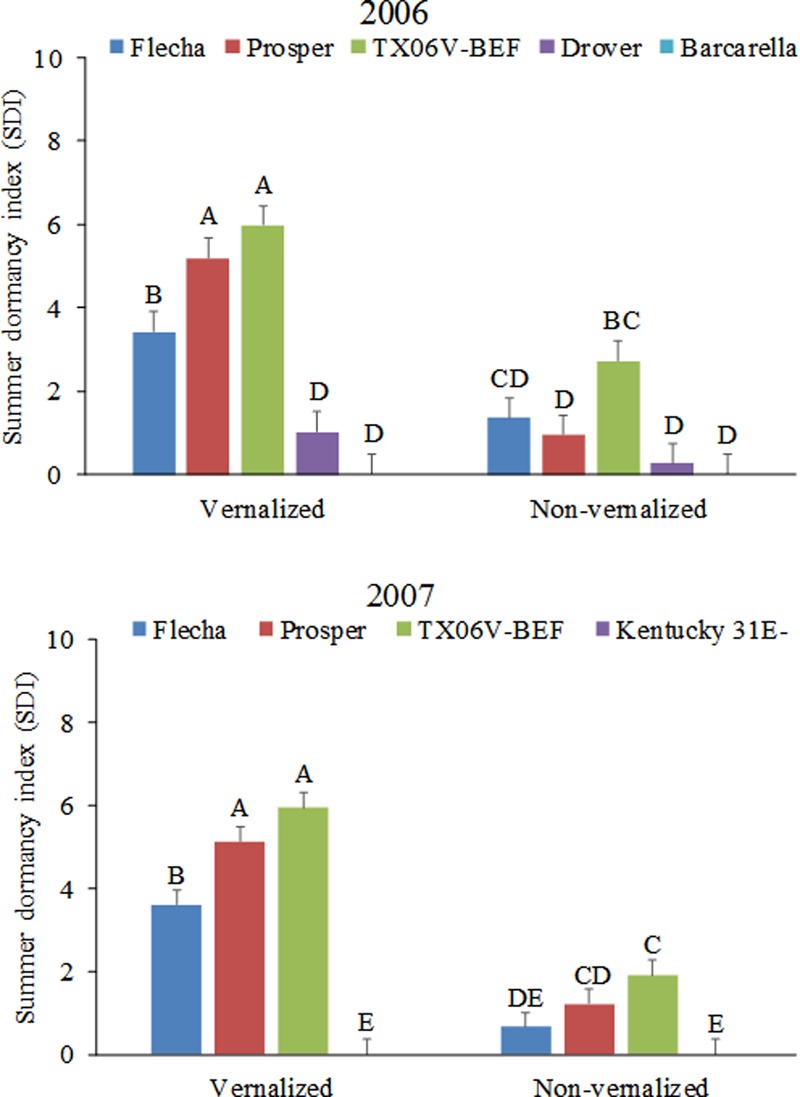
Summer dormancy indexes of tall fescue entries evaluated during summer of 2006 and 2007 as affected by vernalization status. Entries with the same letters are not significantly different. Bars indicate 1 SE.

Vernalization status interacted with tall fescue cultivar in determining the expression of summer dormancy as shown by the calculated SDI (**Figure 4** and **Table 2**). When vernalized, summer-dormant tall fescue cultivars Flecha, Prosper, and TX06V-BEF had higher SDI than summer-active cultivars Drover and Barcarella (2006), and Kentucky 31E- (2007), as expected. Non-vernalized cultivars of tall fescue had lower SDI than vernalized cultivars each year and the difference in expression of summer dormancy was less pronounced among non-vernalized cultivars. Among the summer-dormant cultivars, the experimental line TX06V-BEF and the cultivar Prosper were more summer-dormant (higher SDI) than Flecha when vernalized. The difference was less pronounced in their non-vernalized counterparts.

**Table 2:** The ANOVA results for summer dormancy index (SDI) in the randomized complete block design in experiments conducted during 2006 and 2007 growing seasons.

Variable	Effect	Num DF	Den DF	*F*-value	Pr > F
SDI 2006	Tall fescue variety (C)	4	30	27.61	0.0001
	Vernalization (V)	1	30	46.32	0.0001
	C × V	4	30	6.63	0.0006
SDI 2007	Tall fescue variety (C)	3	24	44.31	0.0001
	Vernalization (V)	1	24	112.75	0.0001
	C × V	3	24	13.44	0.0001

### Potential Involvement of *TFL1/CEN* Gene Family in Summer Dormancy

#### TFL1 Sequence Search, Alignment, and Polymorphism Identification

**FIGURE 5:**
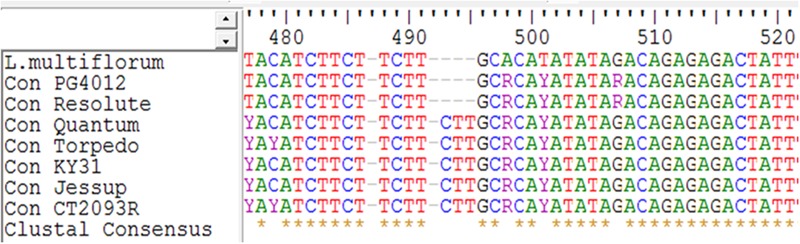
Multiple sequence alignment of the Centroradialis gene sequence from seven tall fescue genotypes and one *Lolium multiflorum*. A unique deletion of three nucleotides at the position 493–496 was present in the known Mediterranean summer-dormant genotypes (Resolute and PG4012) and absent in the continental (KY31, Jessup, and Quantum) and rhizomatous (CT2093R and Torpedo) summer-active genotypes.

Alignment of the *CEN* sequences derived from the seven tall fescue cultivars showed a very high similarity across the ecotypes and even to annual ryegrass. The two Mediterranean summer-dormant types showed 100% similarity to each other and 97% similarity with the continental summer-active genotypes. The continental summer-active genotypes were more similar to the rhizomatous (summer-active) group than the Mediterranean summer-dormant with 99% of the nucleotides being identical (**Table 3**). There was an average of 25 site variations between the Mediterranean summer-dormant genotypes and the continental summer-active, and 28 site variations between the Mediterranean and the rhizomatous genotypes in the *CEN* gene sequence. There are suggestions that Mediterranean and Continental tall fescue trace back to different progenitors. The genomic constitution of Continental tall fescue is believed to be PPG_1_G_1_G_2_G_2_ (Dierking et al., 2015). The P genome originated from the diploid (2n = 2x = 14) meadow fescue (*F. pratensis* Huds.), which is closely related to tall fescue morphologically and the G_1_ and G_2_ genomes are from the tetraploid (2n = 4x = 28) *F. arundinacea* var. *glaucescens* Boiss. Mediterranean tall fescue may have different genome sets derived from different progenitors than Continental tall fescue (Hand et al., 2010). One deletion of three nucleotides at position 493–496 of the *CEN* gene sequence was present in the summer-dormantsummer-dormant tall fescue cultivars but absent in both summer-active continental and rhizomatous cultivars (**Figure 5**). This deletion was also present in the annual ryegrass accession, which is characterized by a determinant growth.

**Table 3:** Matrix of sequence similarity and nucleotide site variations (numbers in parentheses) in the Centroradialis (*CEN*) gene sequences between Mediterranean, continental, and rhizomatous tall fescue genotypes.

Genotypes	*Ecotype*	*L. multiflorum*	PG4012	Resolute	Quantum	Torpedo	KY31	Jessup	CT2093R
*L. multiflorum*		1.00 (0)	0.96 (37)	0.96 (35)	0.95 (43)	0.95 (43)	0.96 (41)	0.96 (41)	0.95 (43)
PG4012	Mediterranean		1.00 (0)	1.00 (2)	0.97 (28)	0.97 (30)	0.97 (27)	0.97 (28)	0.97 (28)
Resolute	Mediterranean			1.00 (0)	0.97 (26)	0.97 (28)	0.97 (25)	0.97 (26)	0.97 (28)
Quantum	Continental				1.00 (0)	0.99 (13)	0.99 (8)	1.00 (2)	0.99 (13)
Torpedo	Rhizomatous					1.00 (0)	0.99 (9)	0.99 (11)	1.00 (0)
KY31	Continental						1.00 (0)	0.99 (6)	0.99 (9)
Jessup	Continental							1.00 (0)	0.99 (11)
CT2093R	Rhizomatous								1.00 (0)

#### Phenotypic Test of Relationship between Determinacy and Summer Dormancy

**FIGURE 6:**
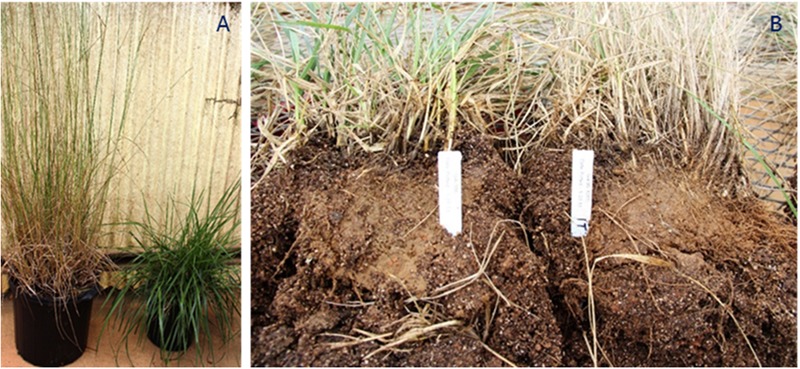
**(A)** Shows two plants of the summer-dormant tall fescue genotype T706-1. The plant on the left was allowed to complete the reproduction cycle and the plant on the right was clipped and prevented from flowering and setting seed. **(B)** Shows two plants of the summer-dormant genotype AGRAFA-126. The plant on the left was clipped frequently and not allowed to flower and produce seeds. The plant on the right was allowed to develop normally, complete the reproduction cycle and produce seeds.

**FIGURE 7:**
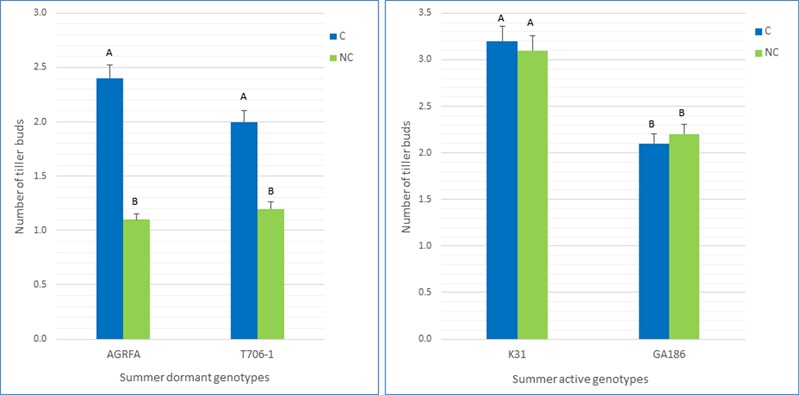
Average number of tiller buds in the summer-dormant genotypes (AGRFA-126 and T706-1) and summer-active (GA186 and Kentucky 31) genotypes. In the non-clipped treatment (NC), the plants were allowed to flower and produce seed. In the clipped treatment (C), the plants were not allowed to flower.

In this experiment, a high number of tiller buds in the basal area indicates more regrowth, and suggests higher summer activity. A low number of buds indicates less regrowth and suggests higher level of summer dormancy (**Figure 6**). The average number of tiller buds in the known summer-dormant genotypes was much higher in the clipped treatment (*P* < 0.01), where the plants were prevented from flowering and setting seed. In the summer-active, non- dormant genotypes, both clipped and non-clipped had the same number (*P* > 0.05) of tiller buds (**Figure 7**).

The average difference in number of tiller buds between clipped and non-clipped treatment groups ranged from -0.1 to 0.1 for the non-summer-dormant genotypes. For the summer-dormant genotypes, the average differences between clipped and non-clipped treatments ranged from 0.8 to 1.2 (**Figure 7**). Fisher’s protected LSD test showed a significant difference (*P* < 0.01) between the average number of buds under clipped and non-clipped treatments of the summer-dormant genotypes T0706-1 and AGRFA (**Figure 7**). There was no significant difference (*P* > 0.05) between clipped treatments and non-clipped treatments of the summer-active cultivars (GA186 and KY31).

## Discussion

### Vernalization and Drought Response in Summer-Active and Summer-Dormant Tall Fescue

The results of these experiments validate the hypothesis that entering the generative stage (flowering) in early spring is required to trigger the onset of dormancy in summer-dormant types of tall fescue and is supported by evidence from studies of *Poa bulbosa* (Ofir and Dorenfield, 1992). Vernalization is required for these cool season grasses in order to initiate flowering, and consequently is a potential player in summer dormancy mechanism. Non-vernalized plants in this study, either did not produce flowering tillers or the number of flowering tillers was not affected by soil moisture deficit. Ofir and Koller (1974) showed that flowering and associated bulb production was a prerequisite for induction of summer dormancy in *Hordeum bulbosum*. Summer-dormant plants that did not undergo vernalization had lower SDI compared to vernalized summer-dormant plants, regardless of the cultivar and growing season. Norton et al. (2006a; 2006b) evaluated responses of autumn- and spring-sown sward of summer-dormant and summer-active orchardgrass and tall fescue cultivars in southern France to long summer drought, full irrigation over summer, or a drought with a simulated mid-summer storm. The authors observed that autumn-sown Flecha (thus likely vernalized) exhibited summer dormancy, whereas spring-sown Flecha (non-vernalized) had a similar shoot DM production to summer-active cultivar Demeter under non-limited soil water conditions in summer. Similarly, orchardgrass sward planted in spring (non-vernalized) did not produce flowering tillers before the onset of summer drought. Non-vernalized orchardgrass plants expressed only partial summer dormancy when compared with autumn-sown (vernalized) plants.

Vernalization studies in wheat (*Triticum aestivum* L.) and barley (*Hordeum vulgare* L.) showed that the vernalization gene *VRN*3 is heterologous to *Arabidopsis* FLOWERING LOCUS T (FT) and confirmed that the FT genes are responsible for natural allelic variation in vernalization requirement in the two annual grasses (Yan et al., 2006). Initiation of flowering is triggered by change in photoperiod and is regulated by a cascade of genes, including *FT, TFL, CEN* and *CO* (Kim et al., 2008; Danilevskaya et al., 2010), *APETALA1 (AP1)/CAULIFLOWER (CAL)* and *LEAFY* [*LFY* (Komeda, 2004; Blazquez et al., 2006)]. Flowering time (*FT*) is an activator of both *AP1/CAL* and *LFY* (Ruiz-García et al., 1997; Huang et al., 2005) and is repressed by *TFL*, which is a homologue of *FT* but serves an opposite function (Kardailsky et al., 1999; Kobayashi et al., 1999). There are other genes in the *TFL1* family, such as *CEN* which is the closest homolog of *TFL*1 in *Arabidopsis* (Danilevskaya et al., 2010). In *Antirrhinum majus* L., expression of the *CEN* gene maintains the inflorescence meristem after transition to flowering (Bradley et al., 1996; Cremer et al., 2001).

The higher Rb concentration in the tissue of non-vernalized plants of summer-dormant tall fescue than vernalized plants suggests a lower water uptake by roots of summer-dormant vs. summer-active tall fescue. This adds further support to the SDI data in suggesting that summer dormancy is not fully expressed in non-vernalized tall fescue plants. Several changes occur during the onset of summer dormancy including growth cessation, change in expression levels of growth related hormones, senescence, and remobilization of nutrient reserves (Powell, 1987; Millard and Neilsen, 1989; Rohde and Bhalerao, 2007; Schwartz and Amasino, 2013).

The increase in total phenolic concentrations in summer-dormant tall fescue entries by the end of summer suggest that these grasses survive summer drought by protecting the meristems from desiccation and oxidative stress. Studies by Volaire (2002) and Norton et al. (2006a,b) have shown that meristematic tissues of perennial forage grasses are not desiccation tolerant, with the exception of *Poa bulbosa* that appears to be programmed for dehydration tolerance of shoot meristematic tissues. Changes associated with dehydration tolerance in meristematic tissues of perennial grasses include adjustment of osmotic potential, accumulation of water-soluble carbohydrates, expression of dehydrin proteins (Volaire, 1995; Volaire and Thomas, 1995), and accumulation of antioxidants (Blokhina et al., 2003; Malinowski and Belesky, 2006).

Even though the focus of this study is not on fescue-endophyte associations, it is worth noting that higher concentrations of phenolic compounds were also observed during drought in tall fescue plants infected with the mutualistic fungal endophyte *E. cenophiala*. These endophytes are known to confer higher drought stress tolerance to the host plant compared to endophyte free (E^-^) plants (Powell et al., 1994; Malinowski et al., 1998, 2005; Zhou et al., 2003). Endophyte-infected plants of tall fescue (Ayad, 1998; Fike et al., 2001) and perennial ryegrass (Briggs et al., 2013) also express greater activity of superoxide dismutase (SOD) than E^-^ plants. Activity of ascorbate peroxidase, an enzyme associated with oxidative stress, was higher in perennial ryegrass plants infected with the fungal endophyte *E. festucae* var. *lolii* (Leuchtmann et al., 2014) than in E^-^ plants exposed to zinc (Zn) stress, suggesting that the endophyte modified plant metabolism by favoring H_2_O_2_ scavenging throughout the catalase process (Bonnet et al., 2000). Summer-dormant tall fescue does not seem to benefit from endophyte infection in terms of superior tolerance to summer drought when compared with E^-^ plants (Norton et al., 2016). One may speculate that summer-dormant tall fescue and other summer-dormant grasses (Malinowski and Belesky, 2006; Malinowski et al., 2009) may benefit from oxidative protection in terms of meristem survival in a similar way as summer-active endophyte-infected tall fescue. It is not clear what environmental or endogenous variables trigger the metabolic pathway of antioxidant production, but it may be a part of the summer dormancy mechanism. The increase in total phenolic concentrations in summer-dormant tall fescue suggest that these compounds play a role in summer drought survival by protecting the meristems from desiccation and oxidative stress.

### Potential Involvement of *TFL1/CEN* Gene Family in Summer Dormancy

The phenotypic and sequence analysis studies presented here gave further support to our postulation that *TFL1/CEN* genes are potential players in the initiation of summer dormancy. The high similarity (more than 97%) between *Lolium perenne TFL1* and tall fescue *CEN* sequences (**Table 3**) is evidence that the two genes are homologs from the same family. The site variations observed between the tall fescue genotypes is a possible indication that *CEN* is under different selective pressures in the different fescue ecotypes depending on their geographical distribution and habitat (**Table 1**). Terminal flower 1 (*TFL1)/CEN* family are important key regulatory genes involved in the control of flowering time and floral architecture in several different plant species. They are highly conserved across plant species and are thought to function in the maintenance of meristem indeterminacy. Mutations in *tfl1* gene cause a switch from indeterminate into determinate and lead to reduced numbers of flower buds and terminal flowers at the shoot apices (Bradley et al., 1997; Guo et al., 2014). *Arabidopsis* plants overexpressing *LpTFL*1 were significantly delayed in flowering and exhibited dramatic changes in architecture including extensive lateral branching and increased growth of all vegetative organs, suggesting that *LpTFL*1 represses flowering and controls axillary meristem identity in ryegrass (Jensen et al., 2001).

The presence of the three nucleotides deletion in the *CEN* sequences of only the known Mediterranean summer-dormant cultivars and the determinate annual *Lolium multiflorum* is evidence for the likelihood of association of determinacy with summer dormancy (**Figure 5**). In a previous study, we found significant associations between *TFL1* and Constans (*CO*) markers and summer dormancy in tall fescue, suggesting the potential relationship between dormancy and determinacy in summer-dormant cool season grasses (Ding and Missaoui, 2017). Constans is a B-box-containing protein promoting the expression of *FT* (Suárez-López et al., 2001; Valverde et al., 2004). It is well established that these circadian clock regulators are involved in determinacy, an agronomically important trait associated with domestication of crop species (Tian et al., 2010). The ectopic expression of *TFL1*-like genes (*CEN* homologues) in maize (*Zea mays* L.) modifies flowering time and inflorescence architecture through maintenance of the indeterminacy of the vegetative and inflorescence meristems (Danilevskaya et al., 2010).

One of the processes preceding dormancy is reduction of plant growth and the arrest of cell division in meristems. Meristems in determinate plants cease vegetative activity soon after photoperiod-induced floral initiation, and undergo transition to reproductive phase (Sablowski, 2007). In this study, tiller bud numbers from 10 replications of two summer-dormant and two non-dormant summer-active tall fescue genotypes showed significant differences between the plants that were prevented from flowering and completing the reproduction cycle (clipped) and those that completed their reproduction cycle (non-clipped) treatments within the known dormant group (**Figures 6, 7**). There was also variation within each treatment in both groups, which can be explained by the fact that tall fescue is an outcrossing species and therefore heterozygosity and heterogeneity are expected within populations. These findings suggest that the completion of the reproductive cycle is necessary for the initiation of summer dormancy and confirm the involvement of genes controlling flowering in the mechanism of summer dormancy.

## Conclusion

Our results suggest that vernalization is an important factor in the onset of summer dormancy in tall fescue. Non-vernalized tall fescue plants do not exhibit summer dormancy as vernalized plants do and behave more like summer-active types. This can be manifested by continuation of shoot growth and high root activity in water uptake under non-limiting soil water content during summer. Summer dormancy in tall fescue should be tested only in plants that underwent vernalization and are not subjected to water deficit during summer months. Total phenolic concentration in tiller bases does not seem to be dependent on vernalization, but environmental variables may have a major effect on accumulation of phenolic compounds (representing antioxidants) in tiller bases to protect meristems from oxidative stress. Summer dormancy trait has been correlated with superior survival after severe summer droughts in many perennial grass species originating from Mediterranean environments. This trait has potential for improving cultivars able to cope with progressing aridity in many Mediterranean and similar environments due to climatic changes especially pronounced in these regions. Sequence analysis of the *TFL1* homolog *CEN* gene from cultivars belonging to summer-dormantsummer-dormant and summer-activesummer-active tall fescue types in addition to tiller bud growth in dormant and non-dormant plants that were either allowed or not allowed to flower and conclude the reproductive cycle, confirmed that stem determinacy is a major component in the mechanism of summer dormancy. The number of variables identified in these studies as potential players in summer dormancy in tall fescue including vernalization, *TFL1/CEN*, water status, and protection from oxidative stress are a further confirmation that summer dormancy is a quantitative trait controlled by several genes with varying effects and prone to genotype by environment interactions.

## Author Contributions

AM and DM conceived and wrote the manuscript. WP and JK contributed to the data generation and interpretation. All authors approved the final manuscript.

## Conflict of Interest Statement

The authors declare that the research was conducted in the absence of any commercial or financial relationships that could be construed as a potential conflict of interest.
